# Surgery for Recurrent Epithelial Ovarian Cancer

**DOI:** 10.1007/s11912-023-01480-8

**Published:** 2023-12-13

**Authors:** Christina Fotopoulou, Ane Gerda Eriksson, Itai Yagel, Suk-Joon Chang, Myong Cheol Lim

**Affiliations:** 1 Department of Surgery and Cancer, Faculty of Medicine, Imperial College London, London, UK; 2 Department of Gynecological Oncology, Division of Cancer Medicine, The Norwegian Radium Hospital, Oslo University Hospital, Oslo, Norway; 3 Shiba Medical Centre, Tel Aviv, Israel; 4 Division of Gynecologic Oncology, Department of Obstetrics and Gynecology, Ajou University School of Medicine, Suwon, Korea; 5 Center for Gynecologic Cancer and Center for Clinical Trials, National Cancer Center, Goyang, South Korea; 6 Rare & Pediatric Cancer Branch and Immuno-Oncology Branch, Division of Rare and Refractory Cancer, Research Institute, National Cancer Center, Goyang, South Korea; 7 Graduate School of Cancer Science and Policy, National Cancer Center, Goyang, South Korea

**Keywords:** Surgery, Relapse, Secondary, Survival, Progression, Ovarian cancer

## Abstract

**Purpose of Review:**

To review evidence around the value and challenges of surgery for recurrent epithelial ovarian cancer (ROC). Both cytoreductive and palliative aspects will be addressed

**Recent Findings:**

Prospective and retrospective evidence demonstrates a significantly longer remission derived from the combination of surgical and systemic modalities as opposed to systemic treatment alone in carefully selected ROC-patients who have relapsed more than 6 months from the end of their 1st line platinum-based chemotherapy. Nevertheless, this benefit appears to be limited when total macroscopic tumor clearance is not achieved. Selection algorithms to identify optimal surgical candidates are of paramount importance to prevent surgical morbidity without the equivalent oncological benefit. In the palliative setting, the risks and benefits of salvage surgery need to be counterbalanced with the advances of conservative techniques for optimal care.

**Summary:**

Well-defined selection algorithms to identify those who will benefit from surgery in the relapsed setting appear to be the key to oncologic and surgical success.

## Introduction

Surgical cytoreduction is the cornerstone of primary treatment for epithelial ovarian cancer. Prospective and retrospective evidence demonstrates a clear correlation between surgical effort and postoperative residual disease with oncologic outcome and overall patients’ survival and quality of life [1]. The same principle has also been applied to later stages of a patients journey, during the 1st and 2nd recurrence and beyond [2•]. While, however, there has been overwhelming retrospective evidence correlating postoperative residual disease and overall survival in the relapsed setting, there has been significant skepticism regarding the role of tumor-biology in the same context. It has long remained unclear whether it was a more favorable intrinsic biology of the disease that allowed better operability and would anyway have been associated with a better overall survival, as opposed to a more adverse disease profile that would have inevitably resulted in worse oncologic outcome while precluding any type of complete surgical resection [1]. The present review will focus on the latest evidence around the risks, benefits, and limitation of surgery for relapsed ovarian cancer (ROC) and will attempt to identify selection algorithms for optimal stratification of patients.

There are two main types of surgery for ROC, reflecting mainly the aim of the surgical attempt: cytoreductive and palliative surgery. Both aspects of care will be addressed here. While cytoreductive surgery aims at removal of all macroscopic visible disease in an effort to improve remission and ultimately survival in the context of a chronic disease condition, at palliative surgery, the goal is to achieve palliation of symptoms that was failed to be managed conservatively, such as bowel obstruction. In the latter setting, complete tumor removal is in the majority of cases not feasible, and any tumor resection is performed to alleviate symptoms and improve, if possible, patients’ quality of life [3]. The differentiation between these two types of surgery is mostly based on the patients’ symptoms and wishes, the type and duration of their response to prior lines of treatments, current tumor dissemination patterns, and future therapeutic options available.

### Cytoreductive Surgery at 1st Relapse

Cytoreductive surgery for the 1st relapse is commonly defined as “secondary” cytoreductive surgery (SCS). This name includes the assumption that the patient has undergone a previous “primary” cytoreduction and may therefore be misleading for those patients who, for which reason, have not had surgery at initial presentation of the disease—a scenario that the gynecological oncology community has increasingly faced for patients diagnosed during the COVID-19 pandemic [4, 5]. The overwhelming body of evidence regarding secondary cytoreduction is for patients who have undergone surgery at time of initial diagnosis. This will be the focus of this review. Benefits of cytoreduction at relapse for surgery-naïve relapsed patients can only be extrapolated from those other scenarios, common sense, experience, traditions, and the overall patients’ journey.

Data demonstrate that cytoreduction at relapse is of very limited benefit in those patients with a short (< 6 months) remission interval from the end of their 1st line cytotoxic platinum-based treatment [6]. Small patient sub-cohorts with mainly lymph node relapse or progression of initially not resected bulky lymph disease have historically been described to derive some benefit from relapsed surgery even if operated within a remission interval shorter than 6 months [7]. However, these patients are not well defined, and a careful consideration of risks and benefits, as well as non-surgical alternatives, is required to prevent surgical morbidity without the expected oncologic benefit. Overall, patients who have not adequately responded or are refractory to platinum should not be routinely considered for cytoreductive surgery.

Evidence regarding the role of surgery for patients with their 1st relapse is best defined in the platinum sensitive setting. Three prospective randomized trials, the GOG-213 [8], SOC-1 [9], and DESKTOP III [10••], have addressed the value of surgical cytoreduction and have added evidence to the numerous previous retrospective analyses [11, 12]. Due to their randomized design, they were in the position to answer the question of tumor biology versus surgical effort.

The DESKTOP III study was based on the selection of patients according to their AGO-score. The AGO score was developed retrospectively in DESKTOP I and validated prospectively in DESKTOP II. The goal of the AGO score was to identify, with simple clinical criteria, patients for whom complete tumor resection can be achieved and patients who would not benefit or would even be harmed by an invasive intervention [13, 14]. The goal of the AGO score was to identify, with simple clinical criteria, patients for whom complete tumor resection can be achieved and patients who would not benefit or would even be harmed by an invasive intervention [13, 14]. The AGO-score was developed only for platinum-sensitive patients and consists of (1) complete resection at primary surgery (alternatively FIGO I/II) and (2) good performance status (ECOG 0) at relapse and absence or low volume ascites (< 500 ml) at relapse. The AGO score was developed retrospectively in DESKTOP I and validated prospectively in DESKTOP II. It demonstrated the likelihood of achieving a complete resection in up to 75% of patients [8]. The DESKTOP III trial randomized patients with a positive AGO score into secondary cytoreductive surgery followed by platinum-based chemotherapy versus chemotherapy only. The study demonstrated a statistically significant benefit in the primary endpoint of OS favoring the addition of surgery (median OS 53.7 versus 46.0 months; HR 0.75; 95% CI 0.58–0.96) and in PFS (median 18.4 versus 14.0 months; HR 0.66; 95%CI 0.54–0.82). The benefit of surgery was limited to patients with macroscopic complete resection (median OS in patients with complete resection 61.9 months). Postoperative morbidity and mortality were acceptably low (90-day mortality 0.5%) [10••]. Of note, the majority of patients in the DESKTOP III trial had multifocal patterns of relapse or peritoneal carcinosis and some even extra-abdominal lesions.

The Asian equivalent SOC-1 trial [9] had a similar design to the European/UK DESKTOP study, with the main difference being the patients selection score; here, it was iModel, a compilation of FIGO stage, residual disease at primary debulking, length of platinum-free interval, ECOG performance status, CA125 level at recurrence, and presence of ascites. This trial was also positive for PFS (median PFS 17.4 versus 11.9 months; HR = 0.58; 95%CI 0.45–0.74) [9]. The majority of patients in the SOC-1 trial had multifocal tumor dissemination patterns and/or peritoneal carcinosis or extra-abdominal lesions. OS data are still immature, indicating a non-significant OS benefit in favor of the surgery arm (median OS 58.1 months versus 53.9 months; HR 0.82; 95%CI 0.57–1.19) [9].

These two abovementioned studies have demonstrated the significance of cytoreduction also for patients with unfavorable presentation of disease such as high tumor load, extra-abdominal lesions, and presence of peritoneal carcinosis. In the DESKTOP III study, PET CT was not routinely used to determine patterns of relapse and operability. Conventional imaging such as CT or MRI was used, confirming that, for example, non-resectable non-bulky microscopic lymph node disease should probably not play a role in the decision making process regarding operability for these patients—as is valid for patients in the primary setting [17].

The third study, the GOG-213 trial from the USA, failed to reproduce the positive results of the DESKTOP III and SOC-1 trials [8]. Here, the hazard ratio for death in the surgery vs. no surgery arms was 1.29 (95%CI, 0.97–1.72; *P* = 0.08), corresponding to a median OS of 50.6 months and 64.7 months, respectively. Equally, no significant PFS survival benefit was seen in the overall population with a HR for disease progression or death (surgery vs. no surgery) of 0.82 (95% CI, 0.66–1.01; median progression-free survival, 18.9 months and 16.2 months, respectively). However, in the sub-cohort of patients with total macroscopic tumor clearance, the HR for progression was 0.62 (95% CI 0.48–0.80), median 16.2 months vs. 22.4 months in favor of the surgery arm. The lack of OS benefit was noted also for this very favorable cohort. The main difference of the GOG-213 trial as opposed the other 2 studies was that no selection algorithms of recruited patients were followed. Patient enrollment was at the discretion of the treating physicians, and only 5% of patients had peritoneal carcinomatosis [8]. In all three trials, surgical morbidity and mortality as well as long-term quality-of-life measures did not differ between the surgery and non-surgery groups.

Of particular interest are the strongly opposing results regarding the impact of SCS on patients’ OS between the DESKTOP III and GOG-213 trials. DESKTOP III demonstrated a 43% better OS for patients who underwent complete cytoreductive surgery at relapse compared to chemotherapy alone. GOG-213 not only failed to demonstrated any significant OS difference between the 2 cohorts but also failed to do so even when considering only patients where complete cytoreduction was achieved. Various hypothesis have been suggested to explain these discrepant results. The most prevailing one is probably the lack of a well-defined selection algorithm of surgical candidates in the GOG-213 study, emphasizing the importance of validated tools and algorithms to preoperatively identify patients who can achieve complete cytoreduction.

Despite the discrepancy in OS benefit of secondary cytoreduction between trials, all 3 prospective randomized studies have consistently demonstrated that patients who undergo complete tumor resection at relapse have a significantly longer PFS than those treated with chemotherapy alone, confirming the value of cytoreductive surgery as part of the treatment package for patients at relapse [8, 9, 10••].

A further consistency in all three trials was that patients underwent secondary cytoreduction first, followed by systemic chemotherapy [8, 9, 10••]. There is no evidence for the oncologic safety or efficacy of the neoadjuvant concept in the relapsed setting, and this should not be part of the routine practice outside of clinical trials.

As the AGO score and the iModel were evaluated in times when primary cytoreductive surgery was standard of care for the majority of patients, the number of patients with successful interval cytoreductive surgery in both scores was limited. The question remained open, whether the scores could be applied to patients with complete resection at interval cytoreductive surgery. Bizzari et al. evaluated the impact of secondary cytoreductive surgery in platinum-sensitive ROC previously treated with neoadjuvant chemotherapy followed by interval cytoreductive surgery and reported similar post-recurrence survival outcomes as for those previously treated with primary cytoreduction, suggesting that current models to select patients for SCS can be safely applied also to patients who were initially operated in the interval setting [15].

A recently published meta-analysis regarding the impact of SCS in platinum-sensitive ROC including 36 studies with 2805 patients from 1983 to 2021 showed that both complete and optimal cytoreductions were associated with improved survival outcomes and as significant moderators in the meta-regression model (*P* < 0.001 and *P* = 0.005, respectively). In the linear regression model, based on 57 studies, the median OS time increased by 9% and 7% when the complete and optimal cytoreduction proportions increased by 10%, respectively, after adjusting for other variables [2•]. Since the meta-analysis included studies from decades ago, where the term “optimal” residual disease was still in use, many of the studies did not differentiate between complete and near complete cytoreduction. Optimal residual disease was defined as 1 cm or 0.5 cm or even 2–2.5 cm depending on the year of publication and country of origin. The conclusion of this large meta-analysis is that SCS, resulting in maximal tumor resection, significantly prolongs OS in women with platinum-sensitive recurrent ovarian cancer. One of the main limitations of the present meta-analysis is that despite the solid statistical efforts to homogenize the large heterogeneity between the studies, generalization to the entire patients’ population is challenging while not all bias are overcome. Moreover, as in any surgical study, largely subjective, non-quantifiable factors such as surgical ability and skills, training, and philosophy/tradition of the surgical team represent unsurpassed bias.

When evaluating specific histological subtypes, such as relapsed low-grade serous ovarian cancer which is an intrinsically more chemoresistance tumor, complete SCS and, to a lesser extent, optimal SCS are associated with improved PFS and OS, irrespective of the platinum-free interval [16]. Interestingly, a short platinum-free interval was not associated with worse survival in this cohort.

### Secondary Cytoreductive Surgery in the Era of Maintenance Therapy

A major challenge is how to incorporate surgical effort at relapse with recent advances in systemic therapy. The majority of studies addressing the value of SCS have been conducted in times where the use of anti-angiogenic agents and PARP-inhibitors was not routine, as it is now. Of note, while the surgical part of the GOG-213 was conducted within a larger study with bevacizumab versus no bevacizumab, only 23% and 5% of the DEKSTOP patients received bevacizumab and PAPR-i, respectively, and equally, only a small proportion of the SOC-1 patients received maintenance therapy during their second-line therapy, presenting a significantly different profile of the three studies [8, 9, 10••]. Hence, the question arises how additional surgical effort can complement the routine implementation of such agents, especially in BRCA mutated/HR-deficient patients.

In the GOG-213 study, the only trial of the three that had anti-angiogenic therapy as standard treatment in one of the arms, all patients who received bevacizumab had similar survival curves regardless of whether they underwent surgery or not, whereas those patients who were operated and opted not to have bevacizumab had a detrimental OS as opposed to those receiving chemotherapy alone [8]. There is no clear explanation for this, but it gives a clear signal that there is an interaction that needs further exploration [8].

### Cytoreduction for the 2nd Relapse and Beyond

Cytoreductive surgery for the 2nd relapse is commonly defined as “tertiary” cytoreductive surgery (TCS). Also here, this name includes the—potentially misleading—assumption that the patient has undergone a previous “primary” and “secondary” cytoreduction, which however is not always the case. As opposed to SCS, there is no prospective evidence to establish survival benefit from TCS, even though retrospective evidence suggests that there is a significant survival benefit in selected patients where complete cytoreduction is achieved even at the 2nd relapse. In the absence of robust and validated selection algorithms for TCS, selection criteria from the secondary setting may be used to guide decision-making to offer surgery in the tertiary setting.

Numerous retrospective multicenter and monocentric analyses have shown that patients with complete cytoreduction in this setting have a longer PFS and OS than patients with residual disease [18–25]. However, none of these studies included direct comparisons with no surgery/chemotherapy alone. The largest retrospective study for tertiary debulking evaluating 406 patients from various cancer centers across the world [24] has demonstrated that, even in the tertiary setting, complete macroscopic tumor clearance significantly improved both overall and progression free survival, also in patients with peritoneal carcinomatosis which failed to retain any prognostic significance on survival after controlling for tumor residual status. The study also identified that the addition of postoperative systemic chemotherapy had a significant impact on overall survival emphasizing the importance of combining systemic and surgical treatment also in this advanced setting.

An ad hoc analysis of the DESKTOP III trial evaluating patients who were randomized in the standard, non-surgical arm and who underwent cytoreductive surgery at a subsequent relapse at investigator’s discretion showed that cytoreductive surgery for subsequent ovarian cancer relapse appears feasible with low mortality in selected patients who received non-surgical treatment at 1st relapse despite a positive AGO-score. This is the highest quality evidence we have so far for the question whether eligible patients who missed the opportunity of potentially life prolonging surgery at 1st relapse would benefit from surgery at the time of their second relapse. Therefore, surgery should be considered as a potential option in carefully selected patients also later in their journey within a specialized gynecological cancer center [26].

### Surgery for Oligometastatic Disease in the Maintenance Era

With the increasing use of novel targeted agents within a maintenance concept, gynecological oncologists are facing also novel, previously uncommon, patterns of relapse. Especially under PAPR-inhibitor maintenance strategies, there are presumed resistant clones of the disease that prevail and progress when the rest of the disease is under control, resulting in oligometastatic tumor relapse patterns. Even though there is no consensus on the exact definition of oligometastatic disease, the term is used to describe a limited metastatic disease, not defined by the size of metastases but rather the number of lesions. The concept of oligometastatic disease is now increasingly more applied in oncology, having been proposed as an intermediate state between localized and disseminated metastatic disease. In the absence of randomized phase 3 trials, early clinical studies also in other tumor-types have suggested improved survival when radical local therapy is added to standard systemic therapy for oligometastatic disease [27]. This concept is also tested for ovarian cancer patients and awakens increasingly the interest of the gynecological oncological community. In operable oligometastatic disease, the same selection and indication rules apply for surgery as outlined above. Still, it is crucial to find the adequate time point to operate versus watch and wait and operate at a later point given the chronic condition of the disease. In surgically not accessible lesions, other non-surgical options can be considered such as stereotactic radiotherapy, cyber knife, and thermal ablation depending on patients’ symptomatology, overall clinical picture, and also, availability of treatment options in each healthcare system [28, 29].

### HIPEC at Cytoreduction for Relapse

There has been only one fully reported prospective randomized phase II trial evaluating the role of HIPEC at SCS [30••]. Patients were intraoperatively randomly assigned to carboplatin HIPEC (800 mg/m^2^ for 90 min) or no HIPEC, followed by five or six cycles of postoperative IV carboplatin-based chemotherapy, respectively. The adequate surgical effort was reflected in the high complete gross resection rates of 82% in the HIPEC- and 94% in the standard-arm patients. Even though the study showed that HIPEC was well tolerated with no perioperative mortality and no difference in use of ostomies, length of stay, or postoperative toxicity, it did not result in superior clinical outcomes. The median PFS in the HIPEC and standard arms was 12.3 and 15.7 months, respectively (hazard ratio, 1.54; 95% CI, 1 to 2.37; *P* = 0.05). There was no significant difference in median overall survival (52.5 vs. 59.7 months, respectively; hazard ratio, 1.39; 95% CI, 0.73 to 2.67; *P* = 0.31).

The findings of this study fit well with those of a recent meta-analysis regarding the value of HIPEC in epithelial ovarian cancer [31] which found that the impact of HIPEC at cytoreductive surgery appears to depend on the timing of the last systemic chemotherapy exposure. No effect was derived from HIPEC in those patients who underwent cytoreduction without prior neoadjuvant chemotherapy. Since in the relapsed setting there is at present no neoadjuvant chemotherapy concept prior to cytoreductive surgery, the role of HIPEC remains limited to clinical trials.

### Palliative Surgery

Advances in the systemic and surgical treatment of ovarian cancer have allowed advanced ovarian cancer patients to live longer after delaying the disease progression during the last decades. However, towards the end of the natural history of this disease, bowel obstruction due to peritoneal carcinomatosis is a common pre-terminal event that causes hospitalization and patients require palliative symptom control to alleviate nausea/vomiting and pain and to improve their quality of life [32].

Bowel obstruction in peritoneally disseminated ovarian cancer constitutes a therapeutic dilemma, since it is most attributed to multilevel stenosis rather than a single point of obstruction making any surgical intervention very challenging. Additionally, novel targeted therapies with anti-angiogenic potential carry a higher risk of fistula formation or intestinal perforation in patients with chronic obstruction symptoms [33].

Management options include surgical treatments, such as bypasses or colostomies/ileostomies, and nonsurgical, conservative therapies, such as bowel decompression, pharmacological management, endoscopic stent placements and percutaneous endoscopic gastrostomies. No randomized trials exist comparing surgical versus medical management, but increasingly more systematic observational studies or even a new cluster randomized study running currently will shed further light in the future.

Retrospective evidence has shown that bowel obstruction surgery at relapse may be associated with higher surgical morbidity and mortality and also high output stomas requiring life-long total parenteral nutrition (TPN) [34, 35]. A recent study [34] from two tertiary ovarian cancer centers of excellence investigating salvage surgery for bowel obstruction demonstrated a mean OS after surgery of 7.8 months; 46% of patients had a residual small bowel length < 180 cm, and 41% of patients were postoperatively in need of life-long TPN. In 80% of patients, a permanent stoma was necessary. Thirty-day morbidity and mortality were 74% and 10%, respectively. Nevertheless, more than 50% of patients were able to receive further courses of chemotherapy after surgery.

Mooney et al. [36] evaluated a cohort of 1518 hospitalized ovarian cancer patients with bowel obstruction. Bowel obstruction at cancer diagnosis (HR = 2.17) and mucinous tumor histology (HR = 1.45) were associated with an increased risk of subsequent obstruction. Surgical management was associated with a lower 30-day mortality (13.4% in women managed surgically vs. 20.2% in women managed non-surgically), but equivalent survival after 30 days and comparable rates of post-obstruction chemotherapy. Median post-obstruction survival was 382 days in women with obstructions of adhesive origin and 93 days in others.

In a recent meta-analysis by Jin et al. of 12 studies involving 2778 cases of bowel obstruction in ovarian cancer, patients had a variety of treatment from surgery (*n* = 1225) and palliative nonsurgical approach (*n* = 1553). The surgery group had a significantly higher remission rate of bowel obstruction (OR = 0.35) but had no manifesting difference in the recurrence rate than the no-surgery group (RR = 0.88). In terms of 30-day mortality rate, the surgery group had a higher mortality rate (RR = 0.45; *P* = 0.000). But surgical treatment can markedly prolong the survival period (HR = 0.33, *P* = 0.000) compared to nonsurgical treatment [37]. There is little doubt that there are inherited bias in this meta-analysis given the patients who were prioritized for surgery over nonsurgical options were certainly the fittest and most favorable over those where not even palliative/salvage surgery was an option.

Surgical intervention should be reserved, when possible, to cases where there is a distal mechanical bowel obstruction and where the formation of a proximal high output small bowel stoma is not likely. Pre-operative imaging demonstrating the most proximal point of bowel obstruction may help identifying patients with a level of obstruction at high risk of iatrogenic short bowel syndrome [35, 38]. Still, since surgical outcome cannot always be fully predicted, and if conservative management has failed, patients need to be counseled regarding the possibility of an iatrogenic short bowel syndrome with all the associated consequences, and appropriate preparations should be made towards this, so that patients can make a fully informed decision. If surgery is planned, the decision-making processes should be led by a gynecological oncologists, since they can evaluate the entire journey of the patient and how this impacts intraoperative decisions. Patients should not be left to be managed by the general surgery, non-expert on call team.

**Fig. 1:**
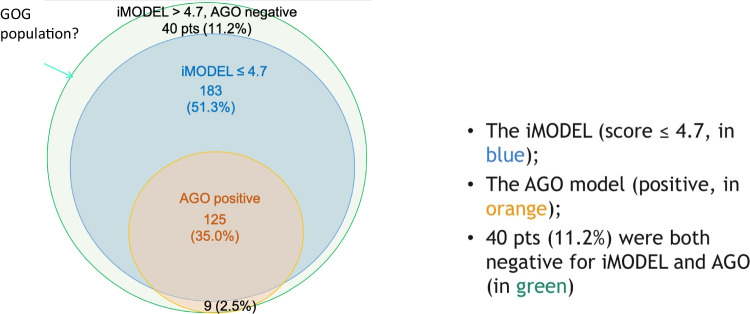
Patient populations and selection scores across the 3 studies

**Fig. 2:**
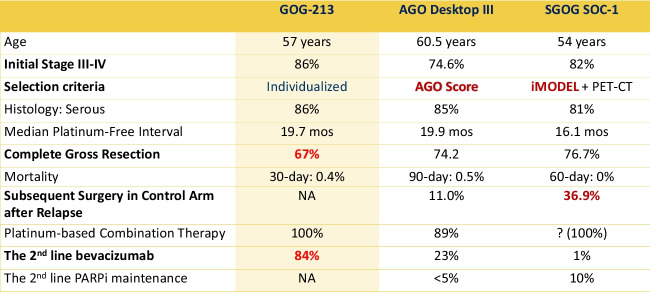
Tumor- and treatment-related characteristics across the 3 studies

**Fig. 3:**
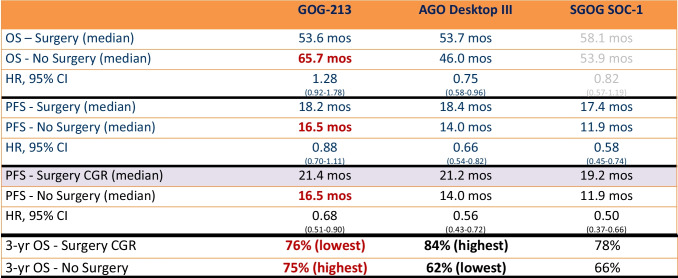
Survival data across the 3 studies

Endoscopic interventional options such as intestinal stents (upper and lower GI-tract), percutaneous endoscopic gastrostomies (see Fig. 1), and pharmacologic management with octreotide acetate, prokinetika, etc. should be prioritized where possible to avoid surgery (39). Anticipated benefits should be carefully balanced against individual risk factors, patients’ co-morbidities, baseline quality of life, previous response to chemotherapy and future systemic options, previous bowel resections, and length of bowel removed already, as well as patient wishes (Figs. 2 and 3).

## Conclusion

Prospective randomized studies have now placed cytoreductive surgery as part of the treatment package also for patients at relapse, following the treatment paradigm in the primary setting of the disease. Still, patients should be aware that the disease will remain chronic even at complete tumor resection and that consolidation with systemic treatment postoperatively will be required. Palliative surgery for bowel-related symptoms such as obstruction should be considered after failure of conservative measures and after careful consideration of the patient’s overall prognosis, quality of life, previous treatments, future therapeutic options, and co-morbidities. Iatrogenic induced short bowel syndrome with the necessity of long life total parenteral nutrition should be avoided, and plans for surgery should be agreed within a multidisciplinary setting.

## References

[1] Hall M, Savvatis K, Nixon K (2019). Maximal-effort cytoreductive surgery for ovarian cancer patients with a high tumor burden: variations in practice and impact on outcome. Ann Surg Oncol.

[2] Baek MH, Park EY, Ha HI, Park SY, Lim MC, Fotopoulou C, Bristow RE (2022). Secondary cytoreductive surgery in platinum-sensitive recurrent ovarian cancer: a meta-analysis. J Clin Oncol.

[3] Colombo N, Sessa C, Bois AD, Ledermann J, McCluggage WG, McNeish I, Morice P, Pignata S, Ray-Coquard I, Vergote I, Baert T, Belaroussi I, Dashora A, Olbrecht S, Planchamp F, Querleu D; ESMO–ESGO Ovarian Cancer Consensus Conference Working Group. ESMO-ESGO consensus conference recommendations on ovarian cancer: pathology and molecular biology, early and advanced stages, borderline tumours and recurrent disease Int J Gynecol Cancer. 2019: ijgc-2019–000308. 10.1136/ijgc-2019-000308. 10.1136/ijgc-2019-000308 31048403

[4] Fotopoulou C, Khan T, Bracinik J, Glasbey J, Abu-Rustum N, Chiva L, Fagotti A, Fujiwara K, Ghebre R, Gutelkin M, Konney TO, Ng J, Pareja R, Kottayasamy Seenivasagam R, Sehouli J, Surappa STS, Bhangu A, Leung E, Sundar S; CovidSurg Gynecological Cancer Collaborators. Outcomes of gynecologic cancer surgery during the COVID-19 pandemic: an international, multicenter, prospective CovidSurg-Gynecologic Oncology Cancer study Am J Obstet Gynecol 2022: 227(5):735.e1–735.e25. 10.1016/j.ajog.2022.06.052 10.1016/j.ajog.2022.06.052 PMC9242690 35779589

[5] Effect of COVID-19 pandemic lockdowns on planned cancer surgery for 15 tumour types in 61 countries: an international, prospective, cohort study. COVIDSurg Collaborative. Lancet Oncol 2021: 22(11):1507–1517. 10.1016/S1470-2045(21)00493-9 10.1016/S1470-2045(21)00493-9 PMC8492020 34624250

[6] Bommert M, Harter P, Heitz F, du Bois A (2018). When should surgery be used for recurrent ovarian carcinoma?. Clin Oncol (R Coll Radiol).

[7] Jain V, Debnath S, Sharma A, Kamboj M, Mohanty A, Rawal S (2022). Isolated lymph node recurrence in epithelial ovarian cancer - management and outcome. J Visc Surg.

[8] Coleman RL, Spirtos NM, Enserro D (2019). Secondary surgical cytoreduction for recurrent ovarian cancer. N Engl J Med.

[9] Shi T, Zhu J, Feng Y (2021). Secondary cytoreduction followed by chemotherapy versus chemotherapy alone in platinum-sensitive relapsed ovarian cancer (SOC-1): a multicentre, open-label, randomised, phase 3 trial. Lancet Oncol.

[10] Harter P, Sehouli J, Vergote I, Ferron G, Reuss A, Meier W, Greggi S, Mosgard BJ, Selle F, Guyon F, Pomel C, Lécuru F, Zang R, Avall-Lundqvist E, Kim JW, Ponce J, Raspagliesi F, Kristensen G, Classe JM, Hillemanns P, Jensen P, Hasenburg A, Ghaem-Maghami S, Mirza MR, Lund B, Reinthaller A, Santaballa A, Olaitan A, Hilpert F, du Bois A (2021). DESKTOP III Investigators Randomized trial of cytoreductive surgery for relapsed ovarian cancer. N Engl J Med.

[11] Bristow RE, Puri I, Chi DS (2009). Cytoreductive surgery for recurrent ovarian cancer: a meta-analysis. Gynecol Oncol.

[12] Zang RY, Harter P, Chi DS, Sehouli J, Jiang R, Tropé CG, Ayhan A, Cormio G, Xing Y, Wollschlaeger KM, Braicu EI, Rabbitt CA, Oksefjell H, Tian WJ, Fotopoulou C, Pfisterer J, du Bois A, Berek JS (2011). Predictors of survival in patients with recurrent ovarian cancer undergoing secondary cytoreductive surgery based on the pooled analysis of an international collaborative cohort. Br J Cancer.

[13] Harter P, Sehouli J, Reuss A (2011). Prospective validation study of a predictive score for operability of recurrent ovarian cancer: the Multicenter Intergroup Study DESKTOP II. A project of the AGO Kommission OVAR, AGO Study Group, NOGGO, AGO-Austria, and MITO. Int J Gynecol Cancer.

[14] Harter P, du Bois A, Hahmann M, Hasenburg A, Burges A, Loibl S, Gropp M, Huober J, Fink D, Schröder W, Muenstedt K, Schmalfeldt B, Emons G, Pfisterer J, Wollschlaeger K, Meerpohl HG, Breitbach GP, Tanner B (2006). Surgery in recurrent ovarian cancer: the Arbeitsgemeinschaft Gynaekologische Onkologie (AGO) DESKTOP OVAR trial Arbeitsgemeinschaft Gynaekologische Onkologie Ovarian Committee; AGO Ovarian Cancer Study Group. Ann Surg Oncol.

[15] Bizzarri N, Marchetti C, Conte C (2022). The impact of secondary cytoreductive surgery in platinum sensitive recurrent ovarian cancer treated with upfront neoadjuvant chemotherapy and interval debulking surgery. Gynecol Oncol.

[16] Goldberg AM, Kim SR, Fazelzad R (2022). Secondary cytoreductive surgery for recurrent low-grade serous ovarian carcinoma: a systematic review and meta-analysis. Gynecol Oncol.

[17] Harter P, Sehouli J, Lorusso D, Reuss A, Vergote I, Marth C, Kim JW, Raspagliesi F, Lampe B, Aletti G, Meier W, Cibula D, Mustea A, Mahner S, Runnebaum IB, Schmalfeldt B, Burges A, Kimmig R, Scambia G, Greggi S, Hilpert F, Hasenburg A, Hillemanns P, Giorda G, von Leffern I, Schade-Brittinger C, Wagner U, du Bois A (2019). A randomized trial of lymphadenectomy in patients with advanced ovarian neoplasms. N Engl J Med.

[18] Leitao MM, Kardos S, Barakat RR, Chi DS (2004). Tertiary cytoreduction in patients with recurrent ovarian carcinoma. Gynecol Oncol.

[19] Karam AK, Santillan A, Bristow RE, Giuntoli R, Gardner GJ, Cass I, Karlan BY, Li AJ (2007). Tertiary cytoreductive surgery in recurrent ovarian cancer: selection criteria and survival outcome. Gynecol Oncol.

[20] Gultekin M, Velipaşaoğlu M, Aksan G, Dursun P, Dogan NU, Yuce K, Ayhan A (2008). A third evaluation of tertiary cytoreduction. J Surg Oncol.

[21] Shih KK, Chi DS, Barakat RR, Leitao MM (2010). Tertiary cytoreduction in patients with recurrent epithelial ovarian, fallopian tube, or primary peritoneal cancer: an updated series. Gynecol Oncol.

[22] Hızlı D, Boran N, Yılmaz S, Turan T, Altınbaş SK, Celik B, Köse MF. Best predictors of survival outcome after tertiary cytoreduction in patients with recurrent platinum-sensitive epithelial ovarian cancer. Eur J Obstet Gynecol Reprod Biol. 2012;163(1):71–5. 10.1016/j.ejogrb.2012.03.018 10.1016/j.ejogrb.2012.03.018 22480413

[23] Fotopoulou C, Richter R, Braicu IE (2011). Clinical outcome of tertiary surgical cytoreduction in patients with recurrent epithelial ovarian cancer. Ann Surg Oncol.

[24] Fotopoulou C, Zang R, Gultekin M, Cibula D, Ayhan A, Liu D, Richter R, Braicu I, Mahner S, Harter P, Trillsch F, Kumar S, Peiretti M, Dowdy SC, Maggioni A, Trope C, Sehouli,  (2013). Value of tertiary cytoreductive surgery in epithelial ovarian cancer: an international multicenter evaluation. J. Ann Surg Oncol.

[25] Falcone F, Scambia G, Benedetti Panici P, Signorelli M, Cormio G, Giorda G, Bogliolo S, Marinaccio M, Ghezzi F, Rabaiotti E, Breda E, Casella G, Fanfani F, Di Donato V, Leone Roberti Maggiore U, Greggi S (2017). Tertiary cytoreductive surgery in recurrent epithelial ovarian cancer: a multicentre MITO retrospective study. Gynecol Oncol.

[26] Jalid Sehouli, Christina Fotopoulou, Ignace Vergote, Alexander Reuss, Gwenael Ferron, Werner Meier, Stefano Greggi, Berit J. Mosgaard, Frederic Selle, Frederic Guyon, Christophe Pomel, Fabrice Lecuru, Rongyu Zang, Kristina Hellmann, Jae-Weon Kim, Margarita Romeo, Francesco Raspagliesi, Brynhildur Eyjólfsdóttir, Andreas Du Bois, Philipp Harter. Role of cytoreductive surgery for the second ovarian cancer relapse in patients previously treated with chemotherapy alone at first relapse: a subanalysis of the DESKTOP III trial. 10.1200/JCO.2022.40.16_suppl.5520 Journal of Clinical Oncology 40, no. 16_suppl (June 01, 2022) 5520–5520.

[27] Guckenberger M, Lievens Y, Bouma AB (2020). Characterisation and classification of oligometastatic disease: a European Society for Radiotherapy and Oncology and European Organisation for Research and Treatment of Cancer consensus recommendation. The Lancet Oncol.

[28] Lazzari R, Ronchi S, Gandini S, Surgo A, Volpe S, Piperno G, Comi S, Pansini F, Fodor C, Orecchia R, Tomao F, Parma G, Colombo N, Jereczek-Fossa BA (2018). Stereotactic body radiation therapy for oligometastatic ovarian cancer: a step toward a drug holiday. Int J Radiat Oncol Biol Phys.

[29] Macchia G, Lazzari R, Colombo N, Laliscia C, Capelli G, D’Agostino GR, Deodato F, Maranzano E, Ippolito E, Ronchi S, Paiar F, Scorsetti M, Cilla S, Ingargiola R, Huscher A, Cerrotta AM, Fodor A, Vicenzi L, Russo D, Borghesi S, Perrucci E, Pignata S, Aristei C, Morganti AG, Scambia G, Valentini V, Jereczek-Fossa BA, Ferrandina G (2020). A large, multicenter, retrospective study on efficacy and safety of stereotactic body radiotherapy (SBRT) in oligometastatic ovarian cancer (MITO RT1 study): a collaboration of MITO, AIRO GYN, and MaNGO groups. Oncologist.

[30] Zivanovic O, Chi DS, Zhou Q, Iasonos A, Konner JA, Makker V, Grisham RN, Brown AK, Nerenstone S, Diaz JP, Schroeder ED, Langstraat CL, Paroder V, Lakhman Y, Soldan K, Su K, Gardner GJ, Andikyan V, Guo J, Jewell EL, Long Roche K, Troso-Sandoval T, Lichtman SM, Moukarzel LA, Dessources K, Abu-Rustum NR, Aghajanian C, Tew WP, Beumer J, Sonoda Y (2021). O’Cearbhaill RE (2021) Secondary cytoreduction and carboplatin hyperthermic intraperitoneal chemotherapy for platinum-sensitive recurrent ovarian cancer: an MSK team ovary phase II study. J Clin Oncol.

[31] Kim SI, Kim JH, Lee S, Cho H, van Driel WJ, Sonke GS, Bristow RE, Park SY, Fotopoulou C, Gynecol Lim MC., Oncol,  (2022). Hyperthermic intraperitoneal chemotherapy for epithelial ovarian cancer: a meta-analysis. Gynecologic Oncology.

[32] Davidson BA, Moss HA, Kamal AH (2018). Top 10 tips palliative care clinicians should know when caring for patients with ovarian cancer. J Palliat Med.

[33] Burger RA, Brady MF, Bookman MA, Monk BJ, Walker JL, Homesley HD (2014). Risk factors for GI adverse events in a phase III randomized trial of bevacizumab in first-line therapy of advanced ovarian cancer: a Gynecologic Oncology Group Study. J Clin Oncol.

[34] Armbrust R, Chekerov R, Sander S, Biebl M, Chopra S, Krell J, Rinne N, Nixon K, Fotopoulou C, Arch Sehouli J, Obstet Gynecol (2022). Surgery due to mechanical bowel obstruction in relapsed ovarian cancer: clinical and surgical results of a bicentric analysis of 87 patients. Archives Gynecol Obstetrics.

[35] Fotopoulou C, Braicu EI, Kwee SL, Kuhberg M, Richter R, Pietzner K, Feldheiser A, Bahra M, Schmidt SC (2013). Sehouli (2013) Salvage surgery due to bowel obstruction in advanced or relapsed ovarian cancer resulting in short bowel syndrome and long-life total parenteral nutrition: surgical and clinical outcome. J Int J Gynecol Cancer.

[36] Mooney SJ (2013). Bowel obstruction in elderly ovarian cancer patients: a population-based study. Gynecol Oncol.

[37] Jin M, Shen F, Li M, Chen Y (2020). Palliative treatment for bowel obstruction in ovarian cancer: a meta-analysis. Arch Gynecol Obstet.

[38] Fotopoulou C, Haidopoulos D (2020). Extraperitoneal en bloc intestinal resection as palliative surgery for treatment refractory bowel obstruction in ovarian cancer relapse. Int J Gynecol Cancer.

[39] Segev Y, Segev L, Schmidt M, Auslender R, Lavie O (2017). Palliative care in ovarian carcinoma patients-a personalized approach of a team work: a review. Arch Gynecol Obstet.

